# A Flexible Carbon Nanofiber-Modified Multi-Channel Electrode Enables High-Sensitivity Electrochemical Detection of Multiple Neuroactive Molecules

**DOI:** 10.3390/s26175413

**Published:** 2026-08-27

**Authors:** Jiao Zhang, Zhiting Zhang, Chunshan Deng, Xiaojian Li, Ji Dai

**Affiliations:** 1 Department of Biomedical Engineering, Southern University of Science and Technology, Shenzhen 518055, China; 2 School of Life and Health Sciences, Shenzhen University of Advanced Technology, Shenzhen 518107, China; 3 Shenzhen-Hong Kong Institute of Brain Science, Shenzhen Institutes of Advanced Technology, Chinese Academy of Sciences, Shenzhen 518055, China; 4 Guangdong Provincial Key Laboratory of Brain Connectome and Behavior, Shenzhen Institutes of Advanced Technology, Chinese Academy of Sciences, Shenzhen 518055, China; 5 University of Chinese Academy of Sciences, Beijing 100049, China

**Keywords:** MEMS flexible electrodes, carbon-based nanomaterials, electrophoretic deposition, chitosan, neuroactive molecules

## Abstract

**Highlights:**

**What are the main findings?**

- A multi-channel flexible electrode was fabricated for the electrochemical detection of neuroactive molecules.
- The carbon nanofiber-modified electrode delivered well-resolved responses for tyrosine, tryptophan, norepinephrine, melatonin, and tryptamine.

**What is the implication of the main finding?**

- Carbon-based composite layers enable high-performance electrochemical interfaces.
- This study offers a new route to develop flexible universal platforms for electrochemical detection.

**Abstract:**

Electrochemical detection is widely acknowledged as a promising technique for quantifying neuroactive molecules, credited to its low cost, portable hardware, and distinctive ability to enable in vivo real-time dynamic monitoring. However, key hurdles still block the technology’s standardization, commercial clinical translation, and on-site application, including poor biocompatibility at the sensing interface, inadequate mechanical stability, and restricted detection generality. To address these drawbacks, this work introduces a multi-channel flexible microwire electrode array. Here, carbon-based nanomaterials build a high-conductivity internal backbone and an analyte-concentrating sensing surface, while chitosan (CS) supports film formation and boosts biocompatibility. These two composite film layers were modified onto the gold surface of MEMS-fabricated flexible microelectrodes via electrophoretic deposition. At optimized voltage and deposition duration, the carbon nanofiber (CNF)-modified electrode outperformed both nitrogen-doped reduced graphene oxide (NGO) electrodes and unmodified gold electrodes. It delivered strong, well-resolved responses for tyrosine, tryptophan, norepinephrine, melatonin, and tryptamine, with detection limits of 9, 45, 50, 15, and 20 μM, respectively, alongside high reproducibility and promising initial stability. Capitalizing on the CS/CNF-Au electrode’s excellent mechanical and electrical performance, this study verifies that tuned carbon-based composite layers enable high-performance electrochemical interfaces, offering a new route to develop flexible universal detection platforms that integrate both biocompatibility and structural reliability.

## 1. Introduction

Neuroactive molecules form the fundamental chemical basis for intercellular information transmission within the nervous system, encompassing a diverse range of molecular types including neurotransmitters, neuropeptides, and neurohormones. Through intricate biochemical signaling networks, these molecules modulate a wide spectrum of biological processes spanning normal physiological function to pathological development. The tyrosine–catecholamine pathway and tryptophan–indoleamine pathway represent two metabolically interconnected yet functionally distinct networks that together underpin the core chemical mechanisms of neuro-endocrine-immune regulation [1]. The two aromatic amino acids tyrosine and tryptophan act as the respective starting precursors for these two pathways. Under enzymatic catalysis, they are processed to produce key neuroactive products, including norepinephrine, melatonin, and tryptamine, which are broadly implicated in the regulation of core physiological activities such as emotion regulation, sleep–wake cycles, cognitive function, motor control, and stress response [2,3,4,5,6,7,8,9,10,11]. Reliable, precise quantitative measurement of tyrosine, tryptophan, norepinephrine, melatonin, and tryptamine is therefore a critical foundational requirement for analyzing neural circuit function, uncovering the molecular mechanisms of disease, and developing effective clinical intervention strategies.

To date, the detection of neuroactive molecules has established a diverse multi-dimensional technical framework including molecular imaging, optical sensing, electrochemical detection, and separation-based analytical methods. Among these approaches, electrochemical detection has emerged as an indispensable research tool, attributed to its inherent advantages of high detection sensitivity, rapid response kinetics, capability for real-time in situ measurement, low instrumentation cost, and compatibility with device miniaturization [12,13]. Flexible electrodes fabricated via micro-electro-mechanical system (MEMS) technology utilize microscale polymer substrates to achieve outstanding mechanical compliance [14,15], which greatly reduces fretting damage to tissue and immune rejection that plague conventional rigid electrode designs. Furthermore, this manufacturing platform enables high-precision, high-consistency fabrication of microelectrode arrays to meet the requirements of multi-channel parallel detection. When integrated with interface modification strategies based on functional nanomaterials, the electrochemical performance of these devices can be further enhanced.

A critical fundamental limitation in electrochemical sensing lies in the persistent challenge of achieving simultaneous detection of multiple target molecules. Conventionally, selective identification of an individual analyte demands specialized surface functionalization of working electrodes to fabricate a target-specific sensing probe. This strictly one-to-one detection paradigm places stringent requirements on precise device fabrication workflows and introduces substantial technical barriers to the implementation of multiplexed co-detection of multiple chemical compounds. To address this limitation, an alternative approach has emerged: the development of universal probes that interact with multiple target analytes without high intrinsic selectivity, where target-specific identification is instead achieved via post-measurement computational signal decoding. Contemporary progress in the engineering of carbon-based nanomaterials has now made the scalable fabrication of such universal electrochemical sensing probes experimentally and practically feasible.

Carbon-based nanomaterials have accelerated the development of electrochemical sensors toward higher sensitivity and multi-target analysis, due to their exceptional electrical conductivity, large specific surface area, and ease of functional modification [16,17,18,19,20,21,22,23,24,25,26]. Carbon nanofibers (CNFs), which possess an extremely high aspect ratio, can assemble into a three-dimensional conductive network within modified electrode films, facilitating electron transfer kinetics and providing abundant electrochemically active reaction sites. Nitrogen-doped reduced graphene oxide (NGO) introduces a high density of structural defects and catalytically active sites that improve electrocatalytic activity and lower the oxidation overpotential of target analytes under appropriate measurement conditions. Chitosan (CS), a naturally occurring cationic polysaccharide, not only enables effective dispersion of carbon nanomaterials to ensure uniform modified film formation, but also inherently exhibits excellent film-forming ability and biocompatibility. At suitable pH conditions, the amino and hydroxyl groups distributed along CS molecular chains can pre-concentrate analytes containing phenolic hydroxyl groups or indole rings—including tyrosine and tryptophan—via hydrogen bonding or electrostatic interactions, thereby amplifying the detection signal. Electrophoretic deposition (EPD) is an efficient, flexible, and versatile material assembly technique that enables controllable, high-yield assembly of CS and carbon nanomaterial composite films onto electrode surfaces, successfully integrating the biocompatibility of CS with the excellent electrical properties of carbon nanomaterials [27,28,29,30,31,32,33,34,35,36].

In this work, we report the development of a multi-channel flexible microwire electrode array. In this device, CNF and NGO were used to construct a high-conductivity internal framework and an analyte-enriched sensing interface, respectively, with CS coordinating film formation and biocompatibility. Two layers of composite films were sequentially modified onto the surface of MEMS-fabricated flexible microelectrodes via EPD. The resulting sensor exhibited favorable electrochemical responses toward tyrosine, tryptophan, norepinephrine, melatonin, and tryptamine. While the present work has only established that the developed sensor generates independent, non-specific electrochemical signals in response to five distinct neuroactive molecules, it establishes a critical foundational framework to guide future research into flexible, universal sensing platforms that integrate superior biocompatibility and inherent structural stability.

## 2. Materials and Methods

### 2.1. Design and Fabrication of MEMS Flexible Microwire Substrate Electrodes

#### 2.1.1. Design of MEMS Flexible Microwire Electrodes

The MEMS flexible microwire electrode developed in this work comprises a flexible microwire substrate electrode and a surface composite functional film layer. For the structural design of the MEMS flexible microwire substrate electrode, it is necessary to comprehensively account for both the requirements of specific application scenarios and signal detection performance. Key design factors include the overall dimension of the electrode, the diameter of detection sites, the number of recording channels, and operability for practical implementation. To minimize the contact area between the electrode and surrounding biological tissue, this study adopts a “mesh-like” open-cell structural design for the internal interconnect region. Prior research has demonstrated that this porous reticular structure facilitates the circulation of biological interstitial fluid and attenuates the inflammatory immune response triggered by electrode implantation [37].

**Figure 1:**
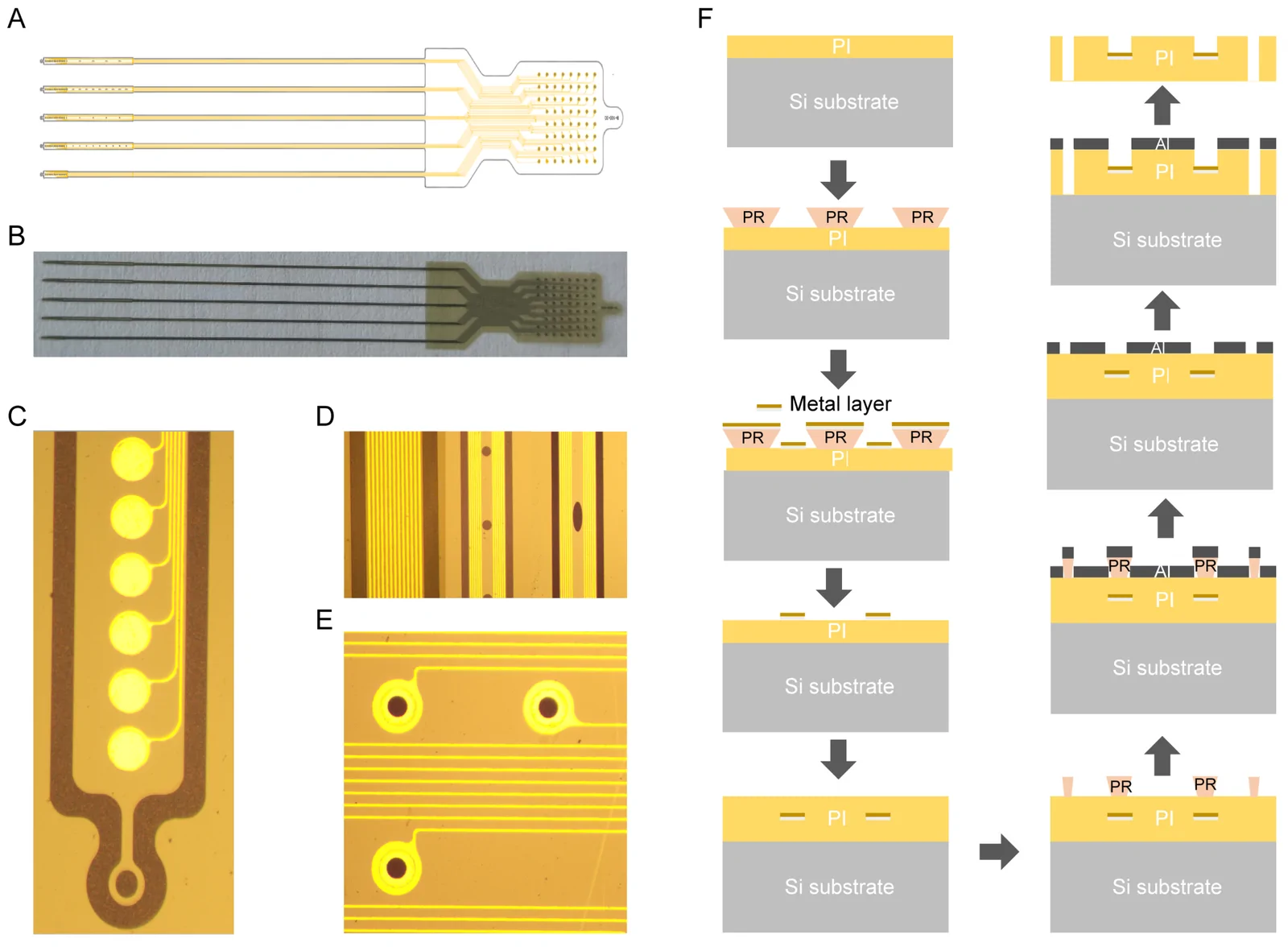
The fabrication of the MEMS flexible microwire substrate electrode. (**A**) Structural design diagram of the electrode; (**B**) overall structural diagram of the electrode; (**C**–**E**) high-magnification microstructural images of the electrode; (**C**) electrode sensing sites and implantation holes; (**D**) long leads of the electrode; (**E**) electrode solder joints. (**F**) The fabrication process of the MEMS flexible substrate electrode. PI: polyimide; Si: silicon; PR: photoresist; Al: aluminum. The metal layer includes a 10 nm thick titanium adhesion layer and a 100 nm thick gold layer.

Structurally, the MEMS flexible substrate electrode designed in this study is divided into three functional regions: the in vivo implantation area, the external interconnect area, and the bonding pad area. The in vivo implantation area integrates the detection sites and internal interconnect traces, and its length is optimized to fully cover the target biological region after implantation while keeping the implantation wound as small as possible. The overall width of the external interconnect area is determined to meet process requirements for trace width and spacing, while its total length is controlled within 1.5~2 mm to preserve sufficient flexibility of the insertion needle and electrode lead during implantation surgery. The size and arrangement of the bonding pad area are matched to the pad layout of the customized flexible printed circuit board, enabling reliable packaging between the electrode and the circuit board via ultrasonic gold ball bonding. In this work, the fabricated MEMS flexible microwire substrate electrode features 61 recording channels, a total thickness of approximately 10 μm, and an inter-site spacing of 30 μm. The full structural design is presented in Figure 1A–E.

The objectives for using multi-channel design are: First, to enable synchronous multipoint detection with high spatial resolution. Multiple microwire probes can be implanted simultaneously into distinct brain regions or different depths of the same region to collect electrochemical signals from multiple sites in synchrony, allowing acquisition of spatial distribution information for neuroactive molecules. This design establishes the required hardware foundation for subsequent in vivo studies focusing on single-time-point chemical imaging across multiple brain regions.

Second, to integrate built-in self-calibration via a multi-channel redundant architecture. During long-term in vivo monitoring, failed recording channels can be accurately identified and eliminated by comparing signal attenuation across adjacent channels, which markedly improves the reliability of collected data. This capability is especially critical for in vivo experiments, where implanted sensors cannot be replaced frequently.

Third, to support both synchronous and independent detection of multiple neural signals. While the current work implements uniform CS/CNF or CS/NGO modification across all electrode channels, the electrochemical discrimination capacity of this single modification layer for five target neuroactive molecules has already been validated, creating a necessary prerequisite for future development of multi-channel differential modification. It is acknowledged that oxidation peaks of different neuroactive molecules may overlap on a uniformly modified electrode; however, the proposed multi-channel architecture allows modification of distinct sensitive membranes or molecular recognition elements on separate channels of the same implant. This enables each channel to exhibit preferential response to specific target molecules, achieving multi-component simultaneous detection at the physical level and eliminating the selectivity challenge caused by peak overlap on single-channel devices. Furthermore, the flexible architecture supports selective modification: only a subset of channels can be functionalized for neurochemical signal acquisition, while the remaining channels are reserved for electrophysiological recording.

#### 2.1.2. Fabrication of MEMS Flexible Microwire Electrodes

The fabrication flow for the MEMS flexible microwire substrate electrode is illustrated in Figure 1F [38]. The process starts with a monocrystalline silicon (Si) wafer (Suzhou Yancai Micro-Nano Technology Co., Ltd., Suzhou, China) serving as a rigid supporting substrate. A 5 μm thick polyimide (PI) polymer (Nantong Jing’ai Microelectronics Technology Co., Ltd., Nantong, China) layer is spin-coated onto the Si wafer and cured at 300 °C to form the flexible base layer of the electrode. The PI layer acts as the structural substrate, providing the electrode with mechanical flexibility and electrical insulation, while the subsequent conductive metal layer is deposited on its surface to form signal transmission paths. Next, an AZ 5214 photoresist (Shenzhen WEINA Electronics Co., Ltd., Shenzhen, China) layer is spin-coated onto the cured PI surface, and microelectrode sites, metal interconnects, and bonding pads are patterned via standard photolithography. The sample is then treated with oxygen plasma to roughen the exposed polymer surface, which improves the interfacial adhesion between the polymer substrate and the subsequently deposited metal layer. A bilayer metal structure, consisting of a 10 nm thick titanium (Ti) adhesion layer and a 100 nm thick gold (Au) conduction layer, is then deposited onto the patterned sample via magnetron sputtering. The sample is subsequently immersed in acetone to lift off excess metal in non-target regions, forming the required conductive metal pattern. Finally, a second 5 μm thick PI layer is coated over the device, and the overall structure of the microelectrode is re-patterned via a second photolithography step. Aluminum (Al) is then used as a hard mask for oxygen reactive ion etching to expose the electrode detection sites, bonding pads, and the peripheral outline of the full device. After chemical removal of the Al hard mask, the completed electrode array is released from the silicon support wafer. The manufacturing scheme adopted in this work relies on standard micro-nano processing technology, which delivers high transferability and repeatability, making it easy to replicate on other manufacturing platforms. The overall architecture and microscale morphology of the as-fabricated electrode are shown in Figure 1A–E.

### 2.2. Functional Modification of MEMS Flexible Microwire Electrodes

#### 2.2.1. Preparation of Composite Solution

A 1% mass-volume fraction CS (Sigma-Aldrich Corporation, St. Louis, MO, USA) solution was prepared using 1% volume fraction aqueous acetic acid (Sinopharm Chemical Reagent Co., Ltd., Shanghai, China) as the solvent, followed by magnetic stirring at room temperature until CS was completely dissolved. CNF (Jiangsu Xianfeng Nanomaterials Technology Co., Ltd., Nanjing, China) was then dispersed into the prepared CS solution at a concentration of 5 mg/mL, with NGO (Jiangsu Xianfeng Nanomaterials Technology Co., Ltd., Nanjing, China) added at a concentration of 0.5 mg/mL, and the mixture was kept under continuous magnetic stirring at room temperature until a homogeneous dispersion was achieved. The resulting composite mixture was heated to 40 °C and stirred magnetically for 30 min, then cooled to room temperature while maintaining stirring. Next, the mixture was treated with an ultrasonic disperser for 20 to 30 min to achieve uniform dispersion of all components, followed by additional ultrasonic degassing for 10 to 20 min to remove dissolved gas and further improve solution uniformity. After that, 0.1 M sodium hydroxide solution (Guangzhou Hewei Pharmaceutical Technology Co., Ltd., Guangzhou, China) was added dropwise to the composite solution to adjust the pH to a range of 5.0–5.8, followed by the addition of genipin (Shanghai Macklin Biochemical Co., Ltd., Shanghai, China) at a mass fraction of 0.2–0.4%. Crosslinking was carried out via magnetic stirring for 24 h at room temperature under dark conditions. Finally, 1 M potassium chloride solution (Guangzhou Hewei Pharmaceutical Technology Co., Ltd., Guangzhou, China) was added to the solution to adjust the conductivity to the target range of 5000–6000 μS/cm.

#### 2.2.2. Electrode Pretreatment and EPD Process for Composite Membrane Layers

First, pretreatment was performed on the electrode: the electrode surface was treated with a vacuum plasma cleaner (Shenzhen Dongxin High-tech Automation Equipment Co., Ltd., Shenzhen, China) for 1–2 min to achieve surface purification and activation. The modified region of the pretreated electrode was then immersed in 0.01 M cysteine solution (Shanghai Macklin Biochemical Co., Ltd., Shanghai, China) and kept static for 24 h under dark conditions to allow the formation of a self-assembled monolayer on the electrode surface. After self-assembly modification was completed, the electrode was soaked and cleaned with anhydrous ethanol (Dongguan Xunye Chemical Reagent Co., Ltd., Dongguan, China) and deionized water for 30 min sequentially to remove excess cysteine physically adsorbed on the surface. Finally, residual moisture on the electrode surface was gently blotted with a dust-free cloth, and the electrode was left to dry at room temperature.

Before starting the EPD experiment, the electrochemical behavior of the base electrode in the CS/CNF composite solution and CS/NGO composite solution was characterized via cyclic voltammetry (CV) to determine the appropriate deposition potential. Chitosan–carbon nanofiber-modified gold (CS/CNF-Au) electrodes and chitosan–nitrogen-doped reduced graphene oxide-modified gold (CS/NGO-Au) electrodes were then fabricated via potentiostatic deposition at varying deposition times using chronoamperometry (CA). After deposition was completed, the modified electrodes were immersed in phosphate-buffered saline (PBS) buffer solution (Guangzhou Hewei Pharmaceutical Technology Co., Ltd., Guangzhou, China) and deionized water sequentially for 20–30 min each, then heated at 60 °C for 10–20 min.

### 2.3. Performance Characterization of MEMS Flexible Microwire Electrodes

To systematically evaluate the performance of MEMS flexible microwire electrodes after surface functional modification, three categories of analysis were conducted: material characterization, fundamental electrochemical characterization, and response characterization of target analytes.

#### 2.3.1. Material Characterization

To validate the feasibility of using EPD to fabricate CS/CNF- and CS/NGO-composite-modified MEMS flexible microwire substrate electrodes, scanning electron microscopy (SEM) was employed to characterize the surface morphology of bare gold (Bare-Au), CS/CNF-Au, and CS/NGO-Au electrodes. Energy-dispersive X-ray spectroscopy (EDX) was further applied to analyze the elemental distribution of the deposited composite films. Raman spectroscopy was used to strengthen the reliability of the characterization of CS/CNF-Au and CS/NGO-Au composites.

#### 2.3.2. Fundamental Electrochemical Characterization

To systematically characterize the electrochemical properties of the three electrode configurations, electrochemical window testing was first performed for all electrodes in 0.1 M potassium chloride solution (Guangzhou Hewei Pharmaceutical Technology Co., Ltd., Guangzhou, China). Electrochemical impedance spectroscopy (EIS) characterization was then conducted separately in two solution systems: 0.01 M PBS solution and a mixed solution of 0.1 M KCl containing 5 mM K_3_[Fe(CN)_6_]/K_4_[Fe(CN)_6_] (Guangzhou Hewei Pharmaceutical Technology Co., Ltd., Guangzhou, China).

According to the Randles–Ševčík equation, for a reversible electrochemical reaction, the peak current is proportional to the electrochemically active surface area A, as given by:

I_p_ = (2.69 × 10^5^) n^3/2^ A D^1/2^ v^1/2^ C

CV was used to measure the oxidation peak current in the 0.1 M KCl + 5 mM K_3_[Fe(CN)_6_]/K_4_[Fe(CN)_6_] mixed solution to calculate the electrochemically active surface area of each electrode. Based on the characterization results obtained, the composite film configuration with superior performance was selected for further process optimization.

To further optimize the EPD process parameters for CS/CNF-Au electrodes, the control variable method combined with CV was employed to measure oxidation current in the 0.1 M KCl + 5 mM K_3_[Fe(CN)_6_]/K_4_[Fe(CN)_6_] mixed solution. The redox performance of CS/CNF-Au electrodes prepared at different deposition voltages and deposition times was compared to determine the optimal EPD process parameters. For all CV characterization experiments, scanning rates of 0.01, 0.05, 0.1, 0.15, 0.2, 0.25, 0.3, 0.35, 0.4, 0.45, and 0.5 V/s were used.

#### 2.3.3. Response Analysis of Target Analytes

To characterize the detection performance of the optimized CS/CNF-Au electrode for five neuroactive molecules (tyrosine (Beijing Solarbio Science & Technology Co., Ltd., Beijing, China), tryptophan (Shanghai Macklin Biochemical Co.,Ltd., Shanghai, China), norepinephrine (Shanghai Titan Scientific Co., Ltd., Shanghai, China), melatonin (Shanghai Macklin Biochemical Co., Ltd., Shanghai, China), and tryptamine (Shanghai Macklin Biochemical Co., Ltd., Shanghai, China)), CV was first used to investigate the reaction mechanism and characteristic oxidation potentials of each target. Differential pulse voltammetry (DPV) was then applied to test the detection sensitivity of the electrode. Sensitivity calibration was performed by sequentially adding aliquots of high-concentration target stock solution to the background buffer to generate stepwise increases in analyte concentration and recording the corresponding DPV response. When the aforementioned neuroactive molecules undergo oxidation at the electrode interface, their reaction products adsorb onto or passivate the electrode surface to varying degrees. To enable accurate detection of the target analyte at low concentration levels, the electrode must be thoroughly cleaned in a blank solution immediately after each concentration measurement is completed. This cleaning step is implemented to minimize the passivation effect induced by accumulated oxidation products. However, for analyte solutions with high concentrations, conventional surface cleaning protocols fail to completely eliminate this residual passivation interference. As a result, continuous detection conducted at the same measurement position suffers from significant systematic deviations in test results. To eliminate this source of measurement error, a single DPV response was collected at different locations across the same electrode, and the corresponding relative standard deviation (RSD) was calculated to evaluate inter-site reproducibility.

To characterize the long-term stability of the CS/CNF-Au electrode, continuous DPV scanning was performed to track the electrode’s response to tyrosine, and stability was evaluated based on the attenuation trend of the response current.

To confirm that the comparative basic electrochemical performance of CS/CNF-Au- and CS/NGO-Au-modified electrodes is consistent with their comparative detection performance for target analytes, CV was used to conduct a comparative analysis of the five neuroactive molecules under identical preparation and testing conditions.

All electrochemical tests were performed using a conventional three-electrode system, with a platinum counter electrode (Shanghai ChuXi Industrial Co., Ltd., Shanghai, China) and a Ag/AgCl reference electrode (Shanghai ChuXi Industrial Co., Ltd., Shanghai, China). All electrochemical characterizations were conducted using a CHI760E electrochemical workstation (Shanghai Chenhua Instrument Co., Ltd., Shanghai, China).

## 3. Results

### 3.1. Surface Morphology and Elemental Composition of MEMS Flexible Microwire Electrodes

**Figure 2:**
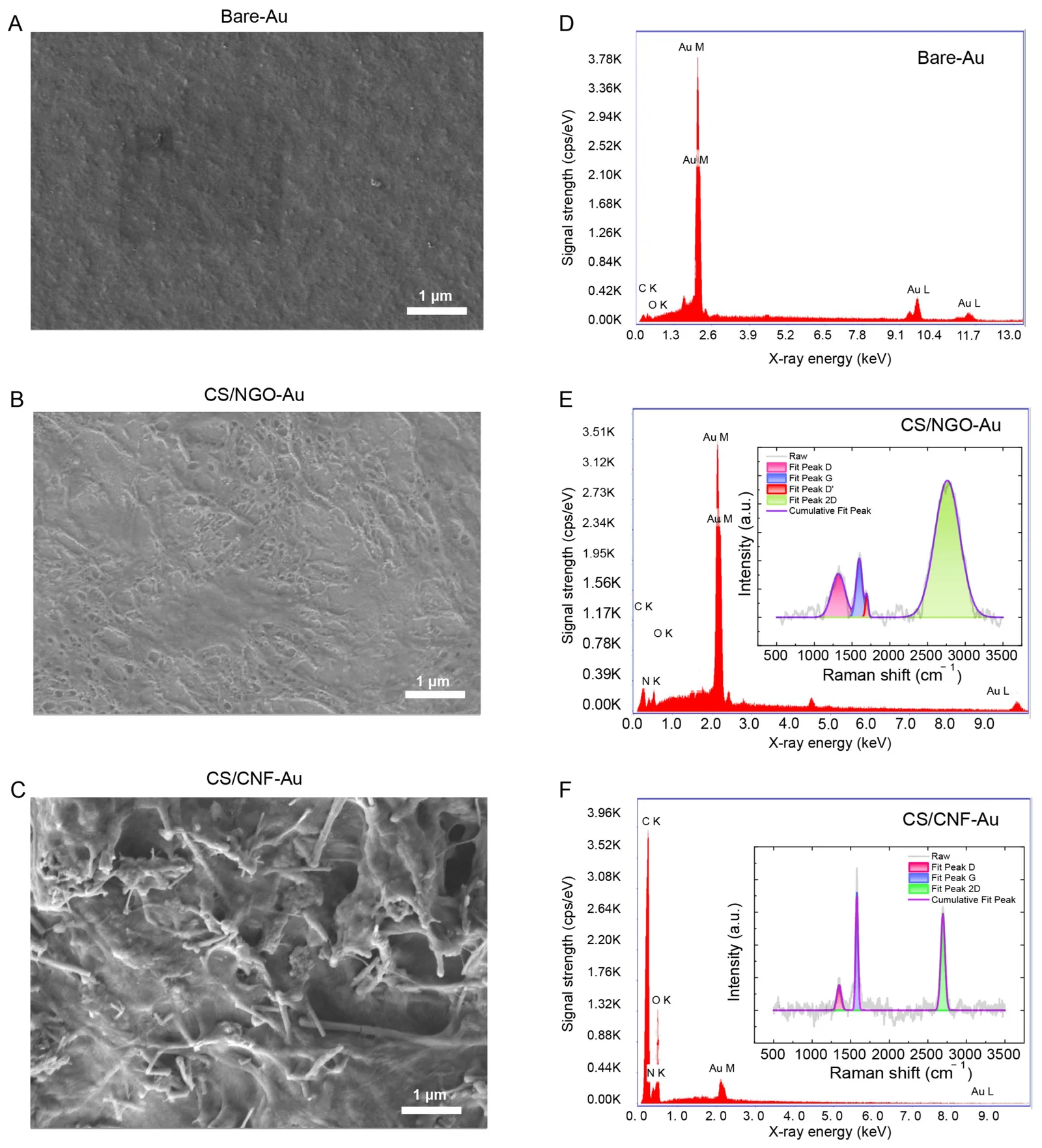
Surface morphology and elemental composition of MEMS flexible microwire electrodes. (**A**–**C**) SEM images of Bare-Au, CS/NGO-Au, and CS/CNF-Au electrodes. (**D**–**F**) EDX analysis of Bare-Au, CS/NGO-Au, and CS/CNF-Au electrodes. In panel (**E**), the inset displays the Raman spectrum of the CS/NGO-Au composite, while the inset provided in panel (**F**) corresponds to the Raman spectrum of CS/CNF-Au.

Firstly, to confirm the successful loading of target materials on the electrode surface and investigate their microstructural features, SEM was employed to characterize the surface morphologies of bare-Au electrodes, CS/NGO-Au electrodes, and CS/CNF-Au electrodes. The bare-Au electrode exhibits a relatively smooth surface morphology (Figure 2A); the CS/NGO-Au electrode displays a two-dimensional planar network structure derived from NGO (Figure 2B); and the CS/CNF-Au electrode shows a three-dimensional conductive network constructed by the interlacing of one-dimensional CNFs (Figure 2C). In contrast to the flat surface of the bare-Au electrode, both the two-dimensional planar network and the three-dimensional interwoven conductive network substantially increase the specific surface area of the electrode, which provides a greater number of active sites for the electrochemical reaction of the target analyte.

EDX was subsequently used to analyze the elemental composition and spatial distribution of the three electrode films. As shown in Figure 2D–F, energy spectrum analysis confirms that the atomic percentages of carbon, nitrogen, and oxygen on the surfaces of CS/NGO-Au and CS/CNF-Au electrodes are markedly higher than those of the bare-Au electrode. Specifically, the proportion of carbon and oxygen atoms on the CS/CNF-Au electrode significantly exceeds that of gold, which further verifies the formation of a dense, certain-thickness CS/CNF composite film on the gold electrode surface.

The Raman spectrum collected from the CS/NGO-Au electrode exhibits distinct characteristic D-band and G-band signals centered at approximately 1350 cm^−1^ and 1580 cm^−1^, respectively. To enable a more precise analysis of structural evolution, multi-peak fitting was conducted on the collected spectrum. The fitting results clearly resolved four distinct characteristic peaks: positioned at ~1350 cm^−1^ (D peak), ~1580 cm^−1^ (G peak), ~1620 cm^−1^ (D’ peak), and ~2700 cm^−1^ (2D peak), with the calculated ID/IG ratio determined to be 1.64. Taken together, these Raman spectroscopic results fully confirm that NGO was successfully incorporated into the CS/NGO-Au electrode, where it retains the layered carbon skeleton structure of the original material and possesses abundant surface defects. The carbon skeleton structure of the CS/CNF-Au electrode was also analyzed via Raman spectroscopy. Its corresponding spectrum displays clear characteristic peaks located near 1350 cm^−1^, 1580 cm^−1^, and 2700 cm^−1^. Following multi-peak fitting of this spectrum, the calculated ID/IG ratio for CS/CNF-Au was determined to be 0.378.

### 3.2. Basic Electrochemical Characterization of MEMS Flexible Microwire Electrodes

Next, the basic electrochemical characterization, including the electrochemical window, EIS, and CV, was tested for bare-Au, CS/NGO-Au, and CS/CNF-Au electrodes.

#### 3.2.1. Electrochemical Window Characterization

**Figure 3:**
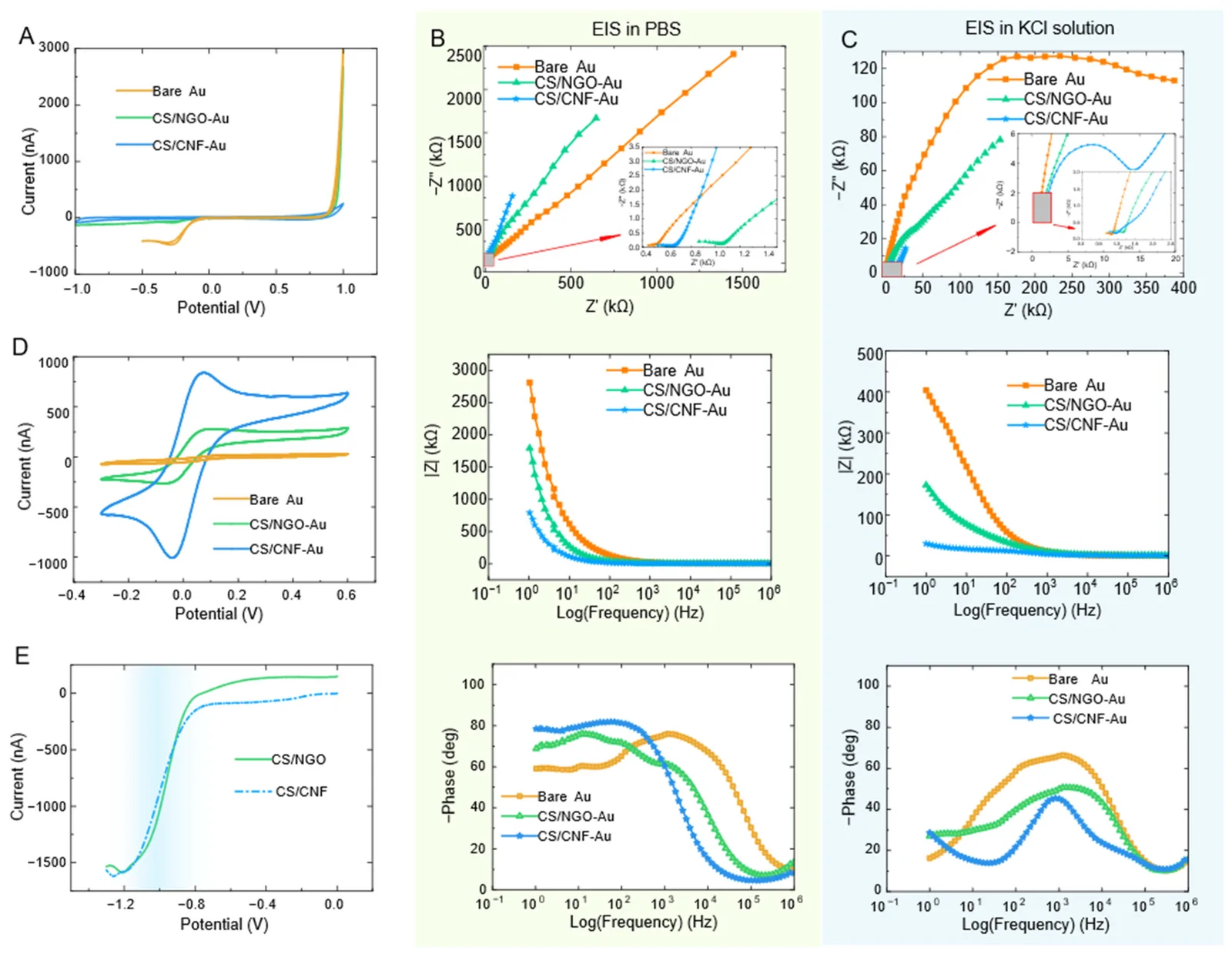
Electrochemical characterization of three types of electrodes. (**A**) Characterization of the electrochemical window for different types of electrodes; (**B**) EIS characterization results in a PBS system without an external redox probe; Row 1: Nyquist plots; Row 2: amplitude–frequency plots; Row 3: phase–frequency plots. (**C**) EIS characterization results in a KCl mixed solution system containing a redox probe; (**D**) electrochemical response results of different electrode interfaces to the [Fe(CN)_6_]^3−^/^4−^ redox probe; (**E**) characterization of the electrochemical behavior of electrodes in different composite solutions.

Electrochemical window testing reveals that the bare-Au electrode possesses the narrowest electrochemical window (Figure 3A), spanning approximately –0.1 V to 0.9 V. Within this potential range, the current response remains stable, exhibiting only the charge–discharge current of the electric double layer with no observable Faradaic reactions, which is consistent with the typical interfacial behavior of metallic electrodes. For the CS/NGO-Au electrode, its electrochemical window is broader than that of bare gold, extending from approximately –1.0 V to 0.9 V. However, during the forward anodic scan to 0.9 V, the oxidation current increases sharply, and the anodic potential limit matches that of the bare-Au electrode.

In comparison, the CS/CNF-Au electrode exhibits the widest electrochemical window, ranging from –1.0 V to 1.0 V, with its voltammetric curve remaining flat across the entire scanned potential range. Even at the high anodic potential of 1.0 V, only a slight increase in oxidation current is detected, with no abrupt current jump observed. No reduction current enhancement is detected in the cathodic region down to –1.0 V. These characteristics confirm that the CS/CNF film has excellent electrochemical stability and an inert interfacial property.

#### 3.2.2. EIS Characterization

To investigate the effect of different carbon nanomaterial modifications on the interfacial properties of gold-based electrodes, EIS technology was used to systematically characterize these electrodes in neutral solution and a KCl-mixed solution.

##### Redox Probe-Free PBS Solution

In the PBS system without the addition of external redox probes, an equivalent circuit model for the modified electrode can be constructed by analyzing the Nyquist plot obtained from EIS characterization (Figure 3B, first row), to elucidate the underlying electrochemical processes of the system. The equivalent circuit is configured as follows: the double-layer capacitance (constant phase element, CPE1), charge transfer resistance (R2, in parallel with CPE1), and Warburg impedance (W1) are connected in series with the solution internal resistance (R1) (Supplementary Figure S1A). This established equivalent circuit accurately describes the electrochemical behavior of both types of modified electrodes and provides a critical reference framework for performance evaluation.

The R2 value derived from EIS fitting results can serve as a quantitative indicator for the reactivity of electrochemical reactions occurring on the electrode surface. EIS analysis demonstrates that the R2 value of the CS/CNF-Au electrode is merely 151.6 Ω, while the R2 value of the CS/NGO-Au electrode reaches 262.3 Ω. It should be emphasized that the unmodified bare gold electrode has a surface property unfavorable for electrochemical reactions, making reliable fitting of EIS data for this type of electrode unfeasible. Nevertheless, the impedance modulus |Z|, as indicated by the amplitude–frequency curves across the 1 Hz to 1000 Hz frequency range (Figure 3B, row 2), follows the order Bare-Au > CS/NGO-Au > CS/CNF-Au. This discrepancy suggests that the CS/CNF-Au electrode has the largest double-layer capacitance.

Phase–frequency curves reflect the frequency dependence of interfacial dynamic processes. In the low-frequency region (1 Hz to ~200 Hz), the CS/CNF-Au electrode exhibits the largest absolute phase angle value (approximately 80°, Figure 3B, row 3), indicating that its interface behavior is closest to that of an ideal capacitor, with a uniform surface structure and minimal energy dissipation. As frequency increases, the relative magnitude of phase angles between different electrodes alternates multiple times. Across 200 Hz to 650 Hz, the CS/NGO-Au electrode exhibits a relatively low absolute phase angle value. In the high-frequency region (above 995 Hz), the bare-Au electrode exhibits the highest absolute phase angle value, likely due to its smooth, non-porous surface.

##### Redox Probe-Containing KCl Mixed Solution

In the KCl mixed solution system containing redox probes, the EIS response primarily characterizes the interfacial electron transfer capability and mass transfer behavior of the modified electrode. Through analysis of the EIS-measured Nyquist plots (Figure 3C, first row), equivalent circuit models were constructed as follows: For the CS/NGO-Au electrode, the equivalent circuit consists of a series connection between the internal resistance (R1) and a parallel combination of the charge transfer resistance (R2) and the double-layer capacitance (CPE1) (Supplementary Figure S1B). For the CS/CNF-Au electrode, the equivalent circuit is structured as a series connection of the internal resistance (R1), the Warburg impedance (W1), and a parallel combination of two branches: one branch is the parallel pair of film resistance (R2) and film capacitance (CPE1), and the other is the parallel pair of charge transfer resistance (R3) and double-layer capacitance (CPE_2) (Supplementary Figure S1C).

Fitting of EIS data yields charge transfer resistance values of 9.384 kΩ for CS/CNF-Au and 28.51 kΩ for CS/NGO-Au. Combined with the EIS fitting results obtained in PBS solution, these findings confirm that the CS/CNF-Au electrode prepared in this work possesses superior electrochemical reactivity. Its lower charge transfer resistance indicates a more favorable electrochemical reaction microenvironment, which facilitates interfacial charge transfer and ultimately improves overall electrochemical performance. Amplitude–frequency curves confirm that across the 1 Hz to 1000 Hz frequency range, the impedance modulus |Z| follows the order Bare-Au > CS/NGO-Au > CS/CNF-Au (Figure 3C, row 2), consistent with the trend observed in probe-free PBS.

However, the phase–frequency curves are largely distinct from those in the PBS solution (Figure 3C, row 3). At the initial low-frequency range (approximately 1 Hz to 1.5 Hz), the CS/CNF-Au electrode exhibits the highest phase angle, followed by the CS/NGO-Au electrode. In the low-frequency transition range (1.5 Hz to 5 Hz), the phase angle of the CS/NGO-Au electrode exceeds that of the CS/CNF-Au electrode. In the mid-to-high frequency range (5 Hz to 1000 Hz), the phase angles of all three electrodes follow a trend of increasing first, then decreasing, with a relaxation peak appearing at approximately 1000 Hz. Among the three electrodes, the CS/CNF-Au electrode has the sharpest peak shape with the lowest peak value.

Overall, the EIS tests demonstrate that the CS/CNF Au electrode has successfully constructed an interface that combines high specific surface area, ideal capacitance behavior, and good electron transfer ability, achieving synergistic optimization of electrochemical activity and interface stability.

#### 3.2.3. CV Characterization

Carried out in 0.1 M KCl solution containing 5 mM K_3_[Fe(CN)_6_]/K_4_[Fe(CN)_6_], the CV testing results demonstrate that the CS/CNF-Au electrode exhibits the most well-defined redox peak shape and the highest peak current (Figure 3D). The CS/NGO-Au electrode performs second, while the bare-Au electrode has the least distinct redox peak and the smallest peak current. Based on the Randles–Ševčík equation, the CS/CNF-Au electrode has the largest electrochemically active surface area. The intermediate peak current of the CS/NGO-Au electrode indicates that NGO also has a high specific surface area, but its effective active area and reaction accessibility are lower than those of CNF. In terms of peak morphology, the CS/CNF-Au electrode shows the clearest peak shape, and the CS/NGO-Au electrode is slightly less defined, in contrast to the indistinct peak shape of the bare-Au electrode.

In addition, carbon nanomaterial modifications also improve the electrochemical response when testing on different scan rates. For the bare-Au electrode (Supplementary Figure S2A), peak current increases with increasing scan rate, but no significant improvement in peak definition is observed. For the CS/CNF-Au and CS/NGO-Au electrodes (Supplementary Figure S2B,C), as the scan rate increases from 0.01 V/s to 0.5 V/s, the redox peak current increases continuously, and the peak shape becomes increasingly distinct.

### 3.3. Optimizing the EPD Process Parameters for MEMS Flexible Microwire Electrodes

Both deposition potential and deposition duration influence the electrochemical activity of carbon nanomaterial-modified electrodes. To identify the optimal parameters, the effect of deposition potential and deposition time was tested independently with the other parameter fixed.

#### 3.3.1. Deposition Potential

Before conducting the potentiostatic deposition test, a CV test was employed to characterize the electrochemical behavior of the working electrode in CS/CNF and CS/NGO composite solutions, aiming to identify the potential range where the reduction current is significantly enhanced. As presented in Figure 3E, the two composite solutions both show robust enhancement when scanning between –1.2 V and –0.8 V. Accordingly, –0.9 V, –1.0 V, and –1.1 V were selected as candidate deposition potentials, and then a systematic investigation into the influence of varying deposition potentials on the electrochemical activity of CS/CNF-Au electrodes was conducted via the control variable method, with deposition time held constant at 300 s.

The experimental outcomes, displayed in Supplementary Figure S3 and summarized in Table 1, demonstrate that under fixed deposition time, a deposition potential of –0.9 V (Supplementary Figure S3A) endows the modified electrode with the highest redox peak current and the most well defined peak shape, with distinguishable redox peaks observable across all tested scan rates. At a deposition potential of –1.0 V (Supplementary Figure S3B), the peak current ranks second, but the resulting peak shape is the broadest of the three conditions. When deposited at –1.1 V (Supplementary Figure S3C), the electrode produces the lowest peak current, though its peak shape is more defined than that obtained at –1.0 V. Based on these results, –0.9 V was selected as the deposition potential for optimizing the deposition time.

#### 3.3.2. Deposition Time

To further optimize the EPD process for CS/CNF-Au electrodes, a systematic analysis of the effect of deposition duration on the electrode’s electrochemical performance was carried out. With the deposition potential fixed at –0.9 V, a series of CS/CNF-Au-modified electrodes were fabricated with deposition durations set to 60 s, 120 s, 300 s, 400 s, and 600 s, respectively. CV characterization was performed on electrodes prepared under each deposition duration, and the resulting voltammetric curves are provided in Supplementary Figure S4.

The experimental results confirm that deposition duration has a pronounced impact on both the response current and peak shape characteristics of the final electrode. The lowest redox current and least distinct peak shape are obtained when deposition is held for 60 s, while a 600 s deposition produces a relatively low current but a more defined peak shape. Moderate deposition durations of 120 s and 400 s yield higher currents and more distinct peak shapes. Among all tested conditions, a 300 s deposition endows the electrode with both the maximum redox current and the clearest peak shape.

### 3.4. Response Characterization of MEMS Flexible Microwire Electrodes to Analytes

Having established the exceptional performance of the CS/CNF-Au electrode through comprehensive fundamental electrochemical characterization, we proceed herein to investigate this electrode’s capability for the detection of neuroactive molecules.

#### 3.4.1. CV and DPV Responses of the CS/CNF-Au Electrode to Five Neuroactive Molecules

**Figure 4:**
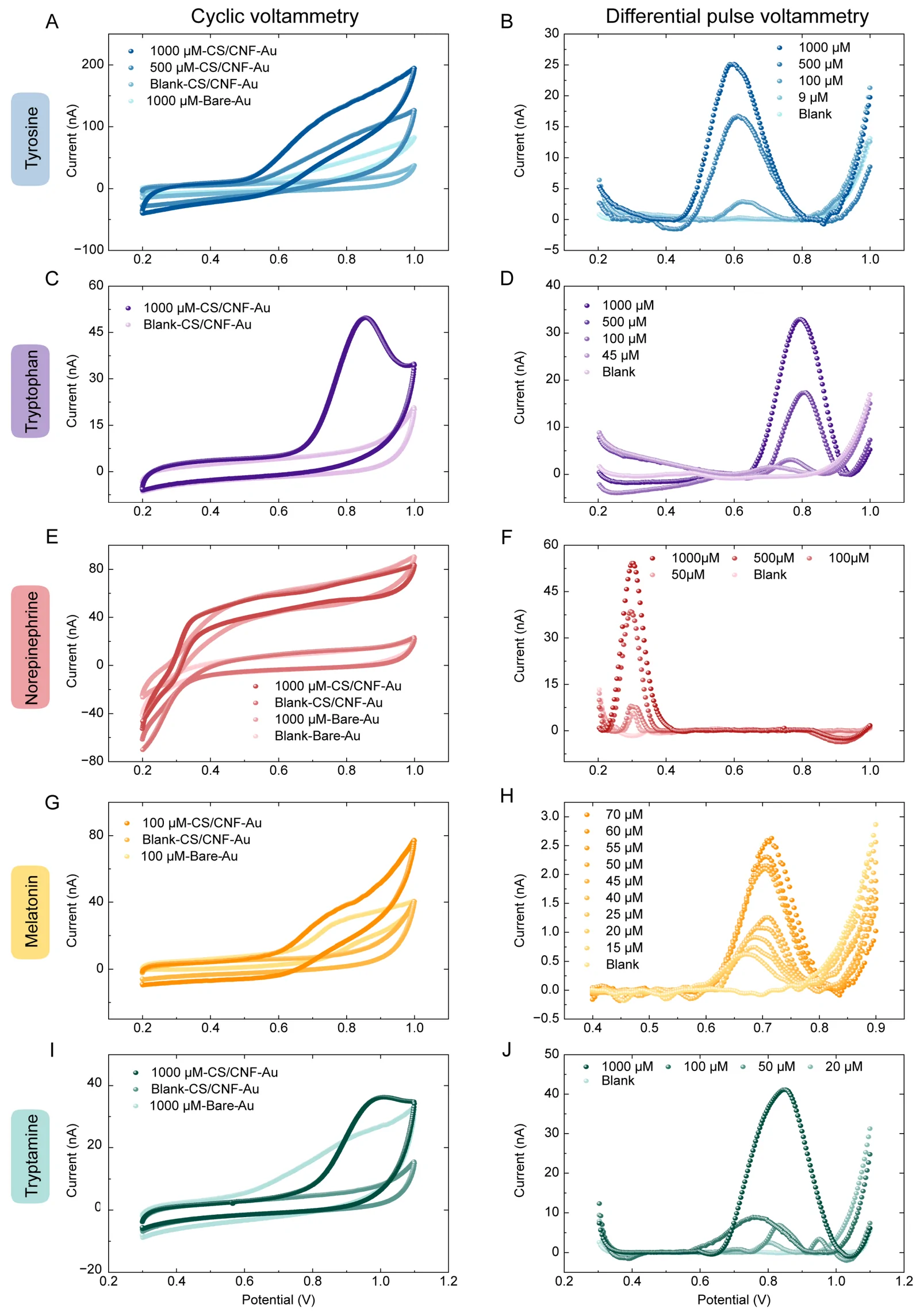
CV and DPV detection results for five neuroactive molecules. (**A**,**B**) Tyrosine; (**C**,**D**) tryptophan; (**E**,**F**) norepinephrine; (**G**,**H**) melatonin; (**I**,**J**) tryptamine; (**A**,**C**,**E**,**G**,**I**) CV measurements; (**B**,**D**,**F**,**H**,**J**) DPV measurements.

CV measurements performed on the CS/CNF-Au electrode for tyrosine detection demonstrate that the oxidation current response increases linearly with increasing tyrosine concentration (Figure 4A). It is important to note that a significant current increase only occurs when the applied potential exceeds 0.55 V. In sharp contrast, the bare-Au electrode produces only a negligible current change even when exposed to a tyrosine concentration as high as 1000 μM. For DPV testing, a clear oxidation peak appears at approximately 0.63 V, and the magnitude of the oxidation current shows a strong positive correlation with tyrosine concentration (Figure 4B).

For tryptophan detection, CV curves show that oxidation current starts to increase at around 0.65 V, with a well-resolved oxidation peak centered at approximately 0.85 V (Figure 4C). In DPV tests, the oxidation peak potential lies between 0.74 V and 0.82 V, and the peak current increases monotonically as tryptophan concentration rises (Figure 4D).

For norepinephrine, CV results indicate that both the CS/CNF-Au electrode and the bare-Au electrode tested at 1000 μM norepinephrine exhibit current values above the background current platform measured in blank solution, yet no obvious redox peaks are identified (Figure 4E). In DPV characterization, a well-defined sharp oxidation peak emerges at approximately 0.3 V, and the current response increases consistently with increasing norepinephrine concentration (Figure 4F).

For melatonin detection, CV tests conducted at 100 μM concentration show that the oxidation current of both the CS/CNF-Au electrode and the bare-Au electrode begins to rise near 0.6 V, but neither electrode produces a distinct oxidation peak. In addition, the current response achieved by the CS/CNF-Au electrode is substantially stronger than that of the bare-Au electrode (Figure 4G). DPV results show that oxidation peak potentials are concentrated in the range of 0.64 V to 0.74 V, with most peaks centered at 0.7 V. The current increases steadily with increasing melatonin concentration, confirming excellent concentration dependence of the response (Figure 4H).

For tryptamine detection, CV curves obtained on the CS/CNF-Au electrode show that oxidation current starts increasing at approximately 0.8 V, accompanied by a distinct oxidation peak. By comparison, the bare-Au electrode reaches a current maximum near 0.6 V but does not form a clear peak shape (Figure 4I). DPV test results show that two oxidation peaks are detected in a 50 μM tryptamine solution, with peak potentials of 0.85 V and 0.95 V, respectively, while only a single oxidation peak is observed at tryptamine concentrations of 20 μM, 100 μM, and 1000 μM (Figure 4J).

Collectively, these results confirm that the CS/CNF-Au electrode delivers superior sensing performance for the detection of tyrosine, tryptophan, norepinephrine, melatonin, and tryptamine.

#### 3.4.2. Reproducibility and Stability Analysis of the CS/CNF-Au Electrode

To further evaluate the detection performance of the CS/CNF-Au electrode fabricated under optimized conditions, DPV was employed to investigate the detection reproducibility (reflecting modification uniformity) for all five neuroactive molecules and the long-term stability for tyrosine detection (Supplementary Figure S5A–E). RSD was used to quantify the DPV responses, and the results are shown in Table 2. Tryptophan and norepinephrine exhibit the best reproducibility in response, followed by tyrosine and tryptamine. Melatonin shows relatively poor reproducibility in peak current response. Stability test results indicate that the oxidation current of tyrosine gradually decreases with repeated scanning, with an attenuation of approximately 39% observed after three consecutive scans, as presented in Supplementary Figure S5F.

#### 3.4.3. Comparing CS/CNF-Au and CS/NGO-Au on Detecting Neuroactive Molecules

Finally, CV was employed to conduct a comparative characterization of the CS/CNF-Au and CS/NGO-Au electrodes. Electrochemical tests were configured with tailored conditions based on analyte properties: measurements for tyrosine, tryptophan, and melatonin were carried out in neutral PBS at pH 7.4; for norepinephrine and tryptamine, tests were performed in acidic acetate-buffered saline (ABS, pH 4.5) (Guangzhou Hewei Pharmaceutical Technology Co., Ltd., Guangzhou, China) under strictly dark conditions to prevent undesired analyte oxidation. All analytes were tested at a fixed concentration of 1000 μM, with the sole exception of melatonin, which was measured at 100 μM due to the high intrinsic sensitivity of the tested electrodes to low concentrations of this compound.

**Figure 5:**
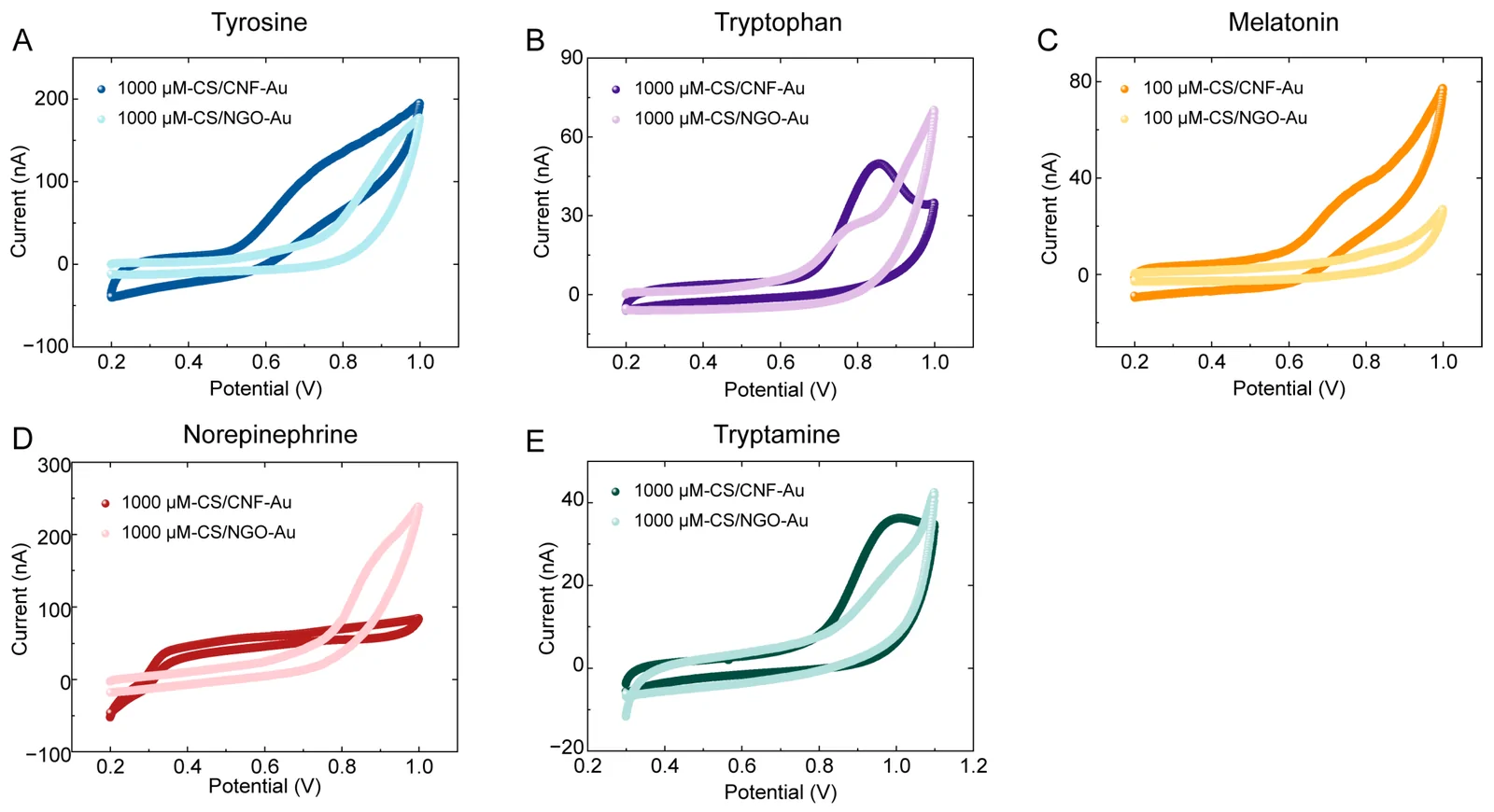
Comparison of CV responses to target compounds using CNF- or NGO-modified electrodes. (**A**) Tyrosine; (**B**) tryptophan; (**C**) melatonin; (**D**) norepinephrine; (**E**) tryptamine.

During tyrosine detection, the oxidation onset potential at the CS/CNF-Au electrode surface is recorded at approximately 0.55 V (Figure 5A). Oxidation current increases steadily as the applied potential rises, producing a broad continuous oxidation current ramp without any well-resolved oxidation peak. In clear contrast, the oxidation onset potential for CS/NGO-Au shifts substantially to 0.75 V, and the resulting response current is drastically attenuated, falling to nearly undetectable levels across the full tested potential window.

For tryptophan detection, the CS/CNF-Au electrode yields a well-resolved irreversible oxidation peak at 0.8 V, with a peak current of approximately 50 nA (Figure 5B). The CS/NGO-Au electrode, however, only exhibits a shallow current response slope near 0.75 V, reaching approximately 30 nA; a subsequent rise to ~70 nA only occurs once the applied potential exceeds 0.85 V.

As a hydrophobic neutral molecule, melatonin demonstrates a sharp rise in oxidation current starting at approximately 0.6 V when measured at the CS/CNF-Au electrode surface (Figure 5C). Again, in contrast, CS/NGO-Au shows no abrupt increase in oxidation current across the full potential range from 0.2 V to 1.0 V; only a slow, steady increase in current is detected, confirming a marked reduction in response sensitivity for this analyte.

The two electrodes exhibit entirely divergent electrochemical behavior for norepinephrine testing (Figure 5D). For CS/CNF-Au, oxidation current increases rapidly between 0.2 V and 0.3 V, but no defined oxidation or reduction peaks form at higher potentials, with only a continuous current ramp observed. In stark opposition, the CS/NGO-Au electrode only shows a steep increase in oxidation current after the applied potential exceeds 0.8 V, and no corresponding reduction peak appears during the reverse scan.

For tryptamine tested under acidic conditions, CS/CNF-Au produces a clear irreversible oxidation peak in the 0.95 V to 1.0 V potential range (Figure 5E), while CS/NGO-Au generates a drastically lower current response across this same potential interval.

Collectively, these comparative results confirm that the CS/CNF-Au electrode offers superior electrochemical detection performance for tyrosine, tryptophan, melatonin, and tryptamine relative to CS/NGO-Au. The only exception to this trend is norepinephrine detection at high potentials, where the CS/NGO-Au electrode outperforms its CS/CNF-Au counterpart.

### 3.5. Anti-Interference Performance of the CS/CNF-Au Electrode

To evaluate the anti-interference capability of the CS/CNF-Au electrode for norepinephrine detection, a verification test was conducted in the presence of norepinephrine and two interfering analytes with similar oxidation potentials: ascorbic acid and uric acid. As shown in Supplementary Figure S6, DPV recorded for the binary mixture of norepinephrine and ascorbic acid exhibits two well-resolved oxidation peaks. These peaks are clearly assigned to the oxidation of norepinephrine at 0.3 V and ascorbic acid at 0.5 V, respectively. When all three analytes (norepinephrine, ascorbic acid, and uric acid) coexist in the test solution, the position and intensity of the norepinephrine oxidation peak remain unchanged. In contrast, the ascorbic acid oxidation peak shows a slight broadening accompanied by an increase in peak current, which arises from the partial overlap of the oxidation peaks of uric acid and ascorbic acid. These findings confirm that the CS/CNF-Au electrode maintains excellent anti-interference performance against ascorbic acid and uric acid during norepinephrine detection.

## 4. Discussion

This study reports the development of a universal electrochemical detection probe capable of detecting five structurally distinct neuroactive small molecules. A major limitation of existing research lies in its predominant focus on highly sensitive single-molecule detection, which fails to address the critical need for multiple target analytes. To overcome this gap, the present work employed a CS/CNF composite film as the sensing interface. Leveraging the high specific surface area and excellent electrical conductivity of carbon nanomaterials, combined with the inherent biocompatibility and superior film-forming properties of CS, this work achieved sensitive detection of five neuroactive molecules: tyrosine, tryptophan, norepinephrine, melatonin, and tryptamine. Although the current findings were obtained from experiments in buffer solutions, this work establishes a foundational framework for subsequent validation in simulated biological matrices. Furthermore, the validation of this probe’s universal applicability provides a novel solution for future implementation of flexible implantable sensors in real-time multi-target monitoring.

### 4.1. Response Analysis of the CS/CNF-Au Electrode to Target Analytes

The CS/CNF-Au electrode fabricated under optimized process parameters exhibited well-defined electrochemical responses to all five analytes, with experimentally determined minimum detectable concentrations of 9 μM for tyrosine, 45 μM for tryptophan, 50 μM for norepinephrine, 15 μM for melatonin, and 20 μM for tryptamine. Differences in the oxidation potential of the five analytes arise primarily from their distinct functional group structures and electronic properties, which provide a fundamental basis for co-detection of multiple molecules.

Furthermore, linear fitting analysis was performed for four analytes: tyrosine, tryptophan, norepinephrine, and melatonin, yielding the corresponding equations: Y = 0.0249X + 0.206 (tyrosine), Y = 0.0329X + 0.54 (tryptophan), Y = 0.0742X + 3.028 (norepinephrine), and Y = 0.0362X + 0.158 (melatonin). The resulting coefficients of determination (R2) are 0.999, 0.999, 0.998, and 0.974, respectively, with corresponding linear concentration ranges of 9–1000 μM, 45–500 μM, 50–500 μM, and 15–100 μM. Employing the standard calibration curve-based statistical method with a preset signal-to-noise ratio of 3.3, the calculated theoretical detection limits for the four analytes are 7.884 μM, 46.371 μM, 49.964 μM, and 13.534 μM, respectively. These calculated values are in close agreement with experimental observations.

For tyrosine, tryptophan, and melatonin, all intercepts of the fitted linear equations fall within a relatively narrow range and exhibit small absolute values, which correspond to an acceptable background signal level. This moderate background intercept can be attributed to the incompletely subtracted non-Faradaic background current. The chitosan/carbon nanofiber-modified gold electrode possesses a large electroactive surface area and high electric double-layer capacitance, which generates a non-negligible charging current or residual background oxidation current in pH 7.4 PBS. Even after blank solution scanning, it remains difficult to achieve complete baseline zeroing. Additionally, the lowest concentration point of the constructed standard curve is not sufficiently low, which introduces a minor extrapolation deviation when extending the calibration curve to the zero-concentration intercept. In contrast, norepinephrine exhibits a significantly larger linear intercept. This deviation may be associated with the acidic supporting electrolyte system used for norepinephrine analysis, the intrinsic physicochemical properties of the norepinephrine molecule, and the specific interaction between the analyte and the modified electrode surface. Notably, DPV testing of tryptamine revealed an unusual concentration-dependent peak behavior: two distinct oxidation peaks appeared at 50 μM, while only a single peak was observed at 20 μM, 100 μM, and 1000 μM (Figure 4J). This phenomenon is hypothesized to originate from the competitive oxidation between adsorbed and freely diffused tryptamine molecules at the electrode surface, combined with the dimerization of oxidation intermediates and subsequent reoxidation of dimers, indicating a concentration-dependent shift in the reaction mechanism. At low concentration (20 μM), the adsorption capacity of tryptamine is limited, and monomer oxidation dominates the reaction, leading to a single oxidation peak. At 50 μM, surface coverage of tryptamine reaches a critical threshold, and the increased concentration of oxidation-generated free radicals promotes dimerization, resulting in the appearance of a second oxidation peak. At high concentrations (100 μM and above), the reaction pathway converges, leading to peak merging and a monotonic increase in peak current with concentration. This observation confirms that the CS/CNF-Au electrode, with its high sensitivity and favorable electron transfer environment, is capable of resolving complex electrochemical reaction mechanisms. It also highlights the necessity of avoiding the concentration range associated with mechanism shifts in practical quantitative analysis to ensure linearity and accuracy.

### 4.2. Reproducibility of Analyte Detection with the CS/CNF-Au Electrode

Reproducibility testing confirmed that the CS/CNF-Au electrode has consistent detection performance, which can be attributed to the high stability of its fabrication process. The CS/CNF composite film deposited via EPD exhibits excellent macroscopic uniformity, with a relatively even distribution of electroactive sites that ensures consistent response across different electrode sites for all target molecules.

Beyond fabrication uniformity, the molecular structure of analytes and their respective oxidation pathways also exert a significant influence on detection reproducibility. Tryptophan and norepinephrine exhibit strong interactions with edge defects and the π-electronic structure on the CNF surface, and their oxidation processes are minimally dependent on microscopic surface heterogeneity, resulting in low response fluctuation between electrode sites. In contrast, the oxidation of tyrosine’s phenolic hydroxyl group readily forms an insulating poly-tyrosine film, and the degree of film formation varies slightly across different electrode sites, leading to moderately larger fluctuations in peak current and potential. Although tryptamine does not exhibit obvious dual peaks at the test concentration (1000 μM), its oxidation intermediates can undergo partial dimerization or non-specific adsorption, processes that are highly sensitive to local surface conditions, resulting in slightly lower peak current reproducibility. For melatonin, the test concentration (100 μM) is one order of magnitude lower than that of the other analytes, which increases the relative contribution of measurement error to the RSD at low concentrations. Additionally, the epoxidation of melatonin’s indole group involves multi-step electron transfer and intermediate adsorption, making the reaction more sensitive to electrode surface microstructure, which further degrades detection reproducibility; instability of melatonin in solution and sampling heterogeneity may also contribute to higher response variation.

Overall, the peak current RSD for all five analytes was below 15%, which meets the accepted reproducibility criterion (RSD ≤ 15%) for trace analysis of biological samples. This confirms that the CS/CNF-Au electrode has a uniformly distributed modification layer and good inter-electrode reproducibility.

### 4.3. Stability of Tyrosine Detection with the CS/CNF-Au Electrode

Stability testing demonstrated that for an irreversible reaction system, such as tyrosine detection, the electrode exhibited 39% current attenuation after repeated testing, which is primarily attributed to surface contamination and passivation. Existing studies confirm that irreversible tyrosine oxidation follows an electrochemical–chemical mechanism: radical intermediates undergo subsequent chemical reactions at the electrode surface to form dimers and insoluble polymers [39], and the resulting insulating polymer film causes progressive signal current decay over time, leading to electrode passivation and contamination [40]. The fast decay rate observed in this study further confirms that the polymerization process occurs rapidly at the electrode surface, which explains the poorly defined tyrosine peak shape observed in CV testing. While this 39% decay rate does not significantly impact short-term single detection experiments, it remains a critical barrier for long-term implantable monitoring applications. Future work will need to incorporate anti-fouling coatings or integrate in situ electrode cleaning strategies to further improve long-term signal stability.

### 4.4. Comparative CV Analysis of Five Neuroactive Molecules at CS/CNF-Au and CS/NGO-Au Electrodes

Under the preparation conditions employed in this work (a CNF concentration of 5 mg/mL and an NGO concentration of 0.5 mg/mL), cyclic voltammetry responses of the two modified electrodes toward five neuroactive molecules were systematically compared. The results reveal that the CS/CNF-Au electrode consistently exhibits lower oxidation overpotential, sharper peak morphology, and a higher signal-to-noise ratio. In contrast, the CS/NGO-Au electrode produces relatively broad peaks alongside a moderately higher background current.

These observed discrepancies are consistent with the findings from earlier basic electrochemical characterization: the CS/CNF-Au electrode possesses the widest electrochemical window, which substantially attenuates background interference originating from solvent electrolysis or oxidation of surface functional groups in the high potential region. Its faster electron transfer kinetics and lower charge transfer resistance create a more unobstructed electron transport pathway for the oxidation of electroactive molecules, while the significantly enlarged electrochemically active surface area provides abundant sites for reactant adsorption and interfacial enrichment.

These performance differences between the two electrodes can be attributed to two contributing factors. On one hand, they stem from the intrinsic differences in dimensionality and electrocatalytic properties between the CNF and NGO nanomaterials. On the other hand, they arise from the higher carbon material loading and greater film thickness of the CS/CNF-modified layer. Consequently, the currently observed accelerated electron transfer kinetics, suppressed background current, and improved peak morphology are at least partially attributable to the denser surface coverage and more complete electrode area utilization enabled by the higher loading. Under the tested conditions, the overall signal-to-noise ratio of the CS/NGO-Au electrode is constrained by its high non-Faradaic background current, which is likely associated with partial exposure of the gold substrate caused by low film coverage. Furthermore, the stacking of NGO nanosheets may lead to a reduction in accessible active area, and the electrocatalytic sites introduced by nitrogen doping have not been fully realized under the current non-optimized loading conditions.

### 4.5. Limitations and Future Prospects

Although this study has achieved phased progress in the preparation and surface functionalization of MEMS-based flexible electrodes, further in-depth exploration and research are still required to enable the practical application of this platform for electrochemical detection of biomolecules in complex biological matrices.

First, systematic in vitro cytotoxicity evaluation and in vivo implantation experiments are needed to comprehensively assess the biocompatibility, host inflammatory response, and long-term functional stability and reliability of CS/CNF and CS/NGO composite membrane materials in real physiological environments. Such work will provide direct experimental evidence supporting the application of these electrodes for in vivo electrochemical detection, which is a critical foundation for advancing their clinical translation.

Second, to address the stability challenges associated with irreversible oxidation molecules such as tyrosine, as well as the issues of complex biological sample composition and non-specific protein adsorption, further optimization of the anti-fouling performance of the electrode interface or development of effective electrode cleaning and regeneration strategies is required.

Third, systematic accelerated aging experiments and long-term cycling tests need to be conducted for both CS/CNF-Au and CS/NGO-Au electrodes. Combining these tests with microscopic characterization will enable in-depth analysis of failure mechanisms such as film delamination and active site saturation, providing clear directions for improving electrode service life and optimizing structural stability.

Fourth, leveraging the established CS/CNF modification platform and integrating artificial intelligence-enabled data processing with advanced signal analysis techniques, this approach is poised to effectively address the selectivity limitations arising from the lack of specific molecular recognition elements in current research. It further offers a novel technical framework to mitigate interference from common electroactive substances in complex biological matrices, including ascorbic acid, uric acid, dopamine, epinephrine, serotonin, and DOPAC, among others.

Currently, the selectivity of CS/CNF-modified electrodes relies primarily on peak potential separation induced by differing electron transfer kinetics of distinct molecules at the carbon nanofiber interface. Nevertheless, this physical separation mechanism remains insufficient for resolving multicomponent systems and complex matrices where peak potentials overlap severely. To date, no systematic interference coexistence experiments have been conducted for this research direction.

The integration of artificial intelligence technologies, such as deep learning and chemometric multivariate curve resolution, can convert this inherently highly universal yet low-selectivity interface into an intelligent sensing platform. Through training neural networks to learn the fine signal characteristics of individual analytes and their mixtures within overlapping voltammetric windows—including peak height, peak shape asymmetry, potential shift, and voltammetric fingerprints—the model is expected to achieve simultaneous discrimination of tyrosine, tryptophan, melatonin, norepinephrine, and tryptamine, without requiring physical separation or specific recognition-based modification. It can also identify and digitally separate the oxidation current contributions of common interferents, including AA, UA, and DA. In this way, selective analyte recognition and computational anti-interference are realized directly at the signal processing level.

For the two CS/carbon nanomaterial composite systems investigated in this work, a systematic investigation of the influence of carbon material loading ratio on composite membrane microstructure and electrochemical performance will further improve our understanding of the structure–activity relationship of these two materials. Finally, leveraging the high-precision and scalable advantages of MEMS technology, future work will explore the development of high-density, high-flexibility multi-channel MEMS flexible electrode arrays and the integration of a multi-functional closed-loop system that combines electrochemical physiological recording and neuromodulation. This will provide a more promising technical platform for basic research and clinical translation in neuroscience.

## 5. Conclusions

In summary, this study proposes a novel controllable fabrication method for CS/CNF- and CS/NGO-composite-film-modified MEMS flexible microelectrode arrays based on EPD technology. This method overcomes the uneven film formation issue associated with traditional drop-coating methods and establishes a correlative mechanism between process parameters, film microstructure, and electrochemical performance, providing a quantitative pathway for high-quality electrode interface modification. This work further constructs an electrochemical detection platform with a universal response to five neuroactive molecules with significant structural differences. By utilizing the high specific surface area and excellent electrical conductivity of carbon nanomaterials, combined with the good biocompatibility of chitosan, this study verifies the potential of flexible sensing platforms for multi-target neuroactive molecule detection. These findings provide new ideas and novel methods for the future development of flexible implantable neurochemical monitoring and closed-loop neuromodulation systems.

## Figures and Tables

**Table 1:** CV characterization data at different deposition potentials.

Electrophoretic Deposition Potential	The Lowest Scan Rate at Which Peaks Are Observable, Along with the Corresponding Peak Potential and Peak Current		Peak Potential and Peak Current at the Highest Scan Rate (0.5 V/s)	
	Oxidation Peak	Reduction Peak	Oxidation Peak	Reduction Peak
–0.9 V	0.092 V 51.44 nA (0.01 V/s)	−0.073 V −64.73 nA (0.01 V/s)	0.093 V 290.1 nA	−0.047 V −326.9 nA
–1.0 V	0.268 V 85.09 nA (0.1 V/s)	0 V −82.74 nA (0.1 V/s)	0.33 V 154.3 nA	−0.034 V −146.2 nA
–1.1 V	0.089 V 27.93 nA (0.05 V/s)	−0.07 V −41.88 nA (0.05 V/s)	0.101 V 90.74 nA	−0.054 V −113.3 nA

**Table 2:** RSD of DPV response results for five neuroactive molecules.

Neuroactive Molecules	RSD	
	Peak Current	Peak Potential
Tyrosine	7.56%	3.25%
Tryptophan	3.97%	0.27%
Norepinephrine	3.25%	0.66%
Melatonin	11.51%	3.23%
Tryptamine	6.55%	0.27%

## Data Availability

The raw data supporting the conclusions of this article will be made available by the authors on request.

## Supplementary Materials

The following supporting information can be downloaded at: https://www.mdpi.com/article/10.3390/s26175413/s1. Figure S1: Equivalent circuit model for EIS fitting; Figure S2: Electrochemical response results of the [Fe(CN)_6_]^3−^/^4−^ redox probe on different electrode interfaces; Figure S3: Electrochemical response results of the CS/CNF-Au electrode pair to the [Fe(CN)_6_]^3−^/^4−^ redox probe at different deposition potentials; Figure S4: Electrochemical response results of CS/CNF-Au electrodes prepared at different deposition times to the [Fe(CN)_6_]^3−^/^4−^ redox probe; Figure S5: Results of reproducibility and stability tests for the CS/CNF-Au electrode; Figure S6. The anti-interference performance of the CS/CNF-Au electrode.

## Abbreviations

The following abbreviations are used in this manuscript:

MEMS	Micro-electro-mechanical system
CNF	Carbon nanofiber
NGO	Nitrogen-doped reduced graphene oxide
CS	Chitosan
EPD	Electrophoretic deposition
CV	Cyclic voltammetry
DPV	Differential pulse voltammetry
SEM	Scanning electron microscopy
EDX	Energy-dispersive X-ray spectroscopy
EIS	Electrochemical impedance spectroscopy
RSD	Relative standard deviation
PBS	Phosphate-buffered saline
