# Supplementary material for: A Flexible Carbon Nanofiber-Modified Multi-Channel Electrode Enables High-Sensitivity Electrochemical Detection of Multiple Neuroactive Molecules
Source: Sensors (Basel). 2026 Aug 27;26(17):5413. doi: 10.3390/s26175413 (PMC13568076; doi:10.3390/s26175413)
Supplement: Supplementary file 1 [file sensors-26-05413-s001.zip › sensors-4417212-supplementary.pdf]

Supplementary Materials

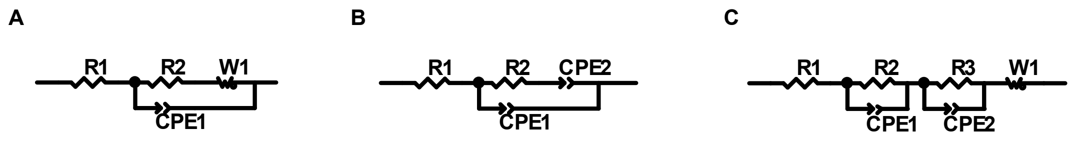

Supplementary Figure S1. Equivalent Circuit Model for EIS Fitting. (A) CS/CNF-Au and CS/NGO-Au electrodes in PBS solution; (B) CS/NGO-Au in KCl solution; (C) CS/CNF-Au in KCl solution.

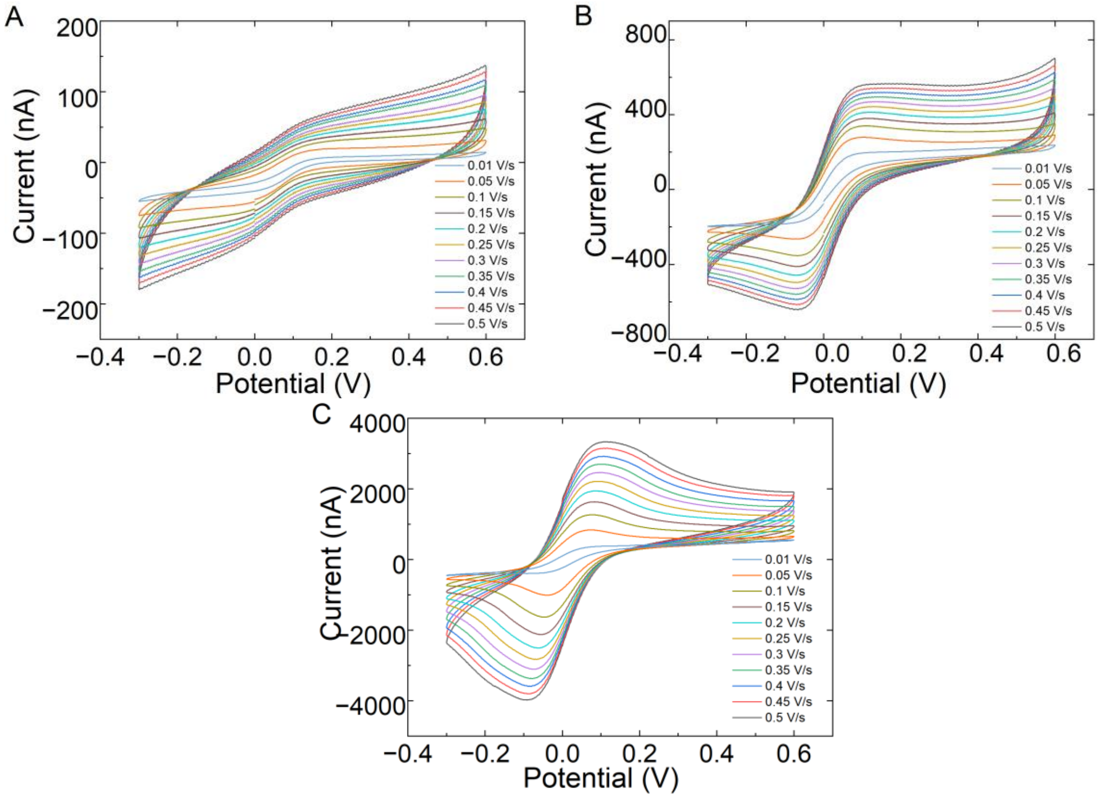

Supplementary Figure S2. Electrochemical response results of the $[Fe(CN)_6]^{3-/4-}$ redox probe on different electrode interfaces. (A) Bare Au; (B) CS/NGO-Au; (C) CS/CNF-Au.

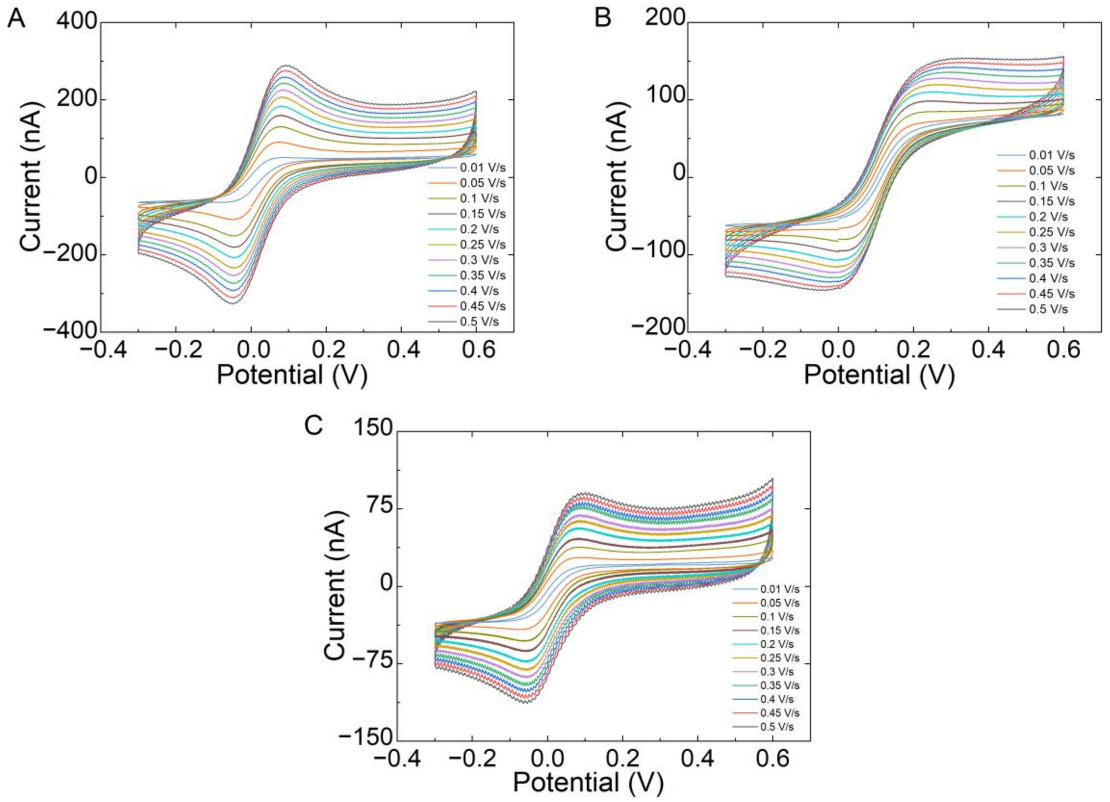

Supplementary Figure S3. Electrochemical response results of the CS/CNF-Au electrode pair to the $[Fe(CN)_6]^{3-/4-}$ redox probe at different deposition potentials. (A) –0.9 V; (B) –1.0 V; (C) –1.1 V.

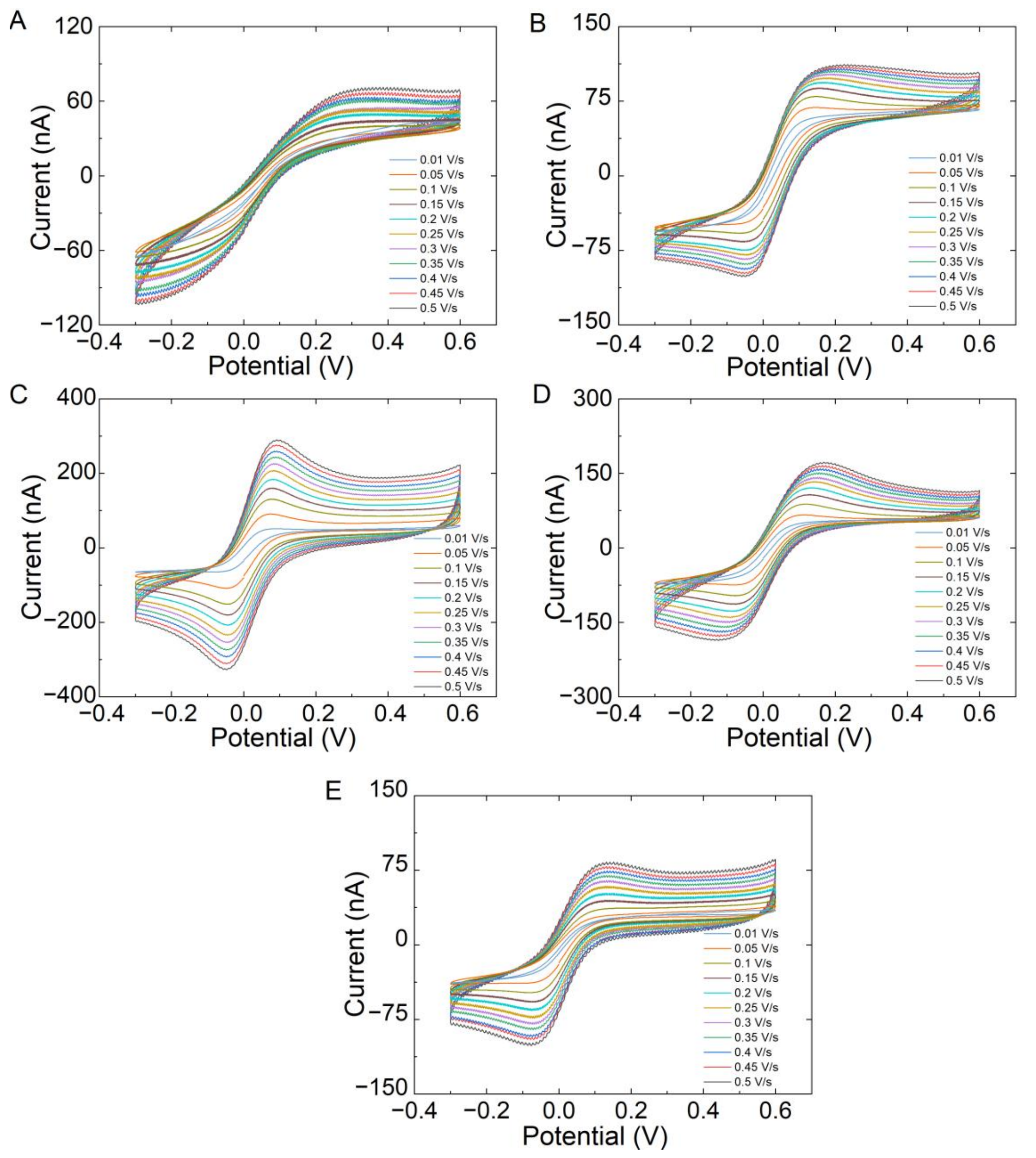

Supplementary Figure S4. Electrochemical response results of CS/CNF-Au electrodes prepared at different deposition times to the $[Fe(CN)_6]^{3-/4-}$ redox probe. (A) 60 s; (B) 120 s; (C) 300 s; (D) 400 s; (E) 600 s.

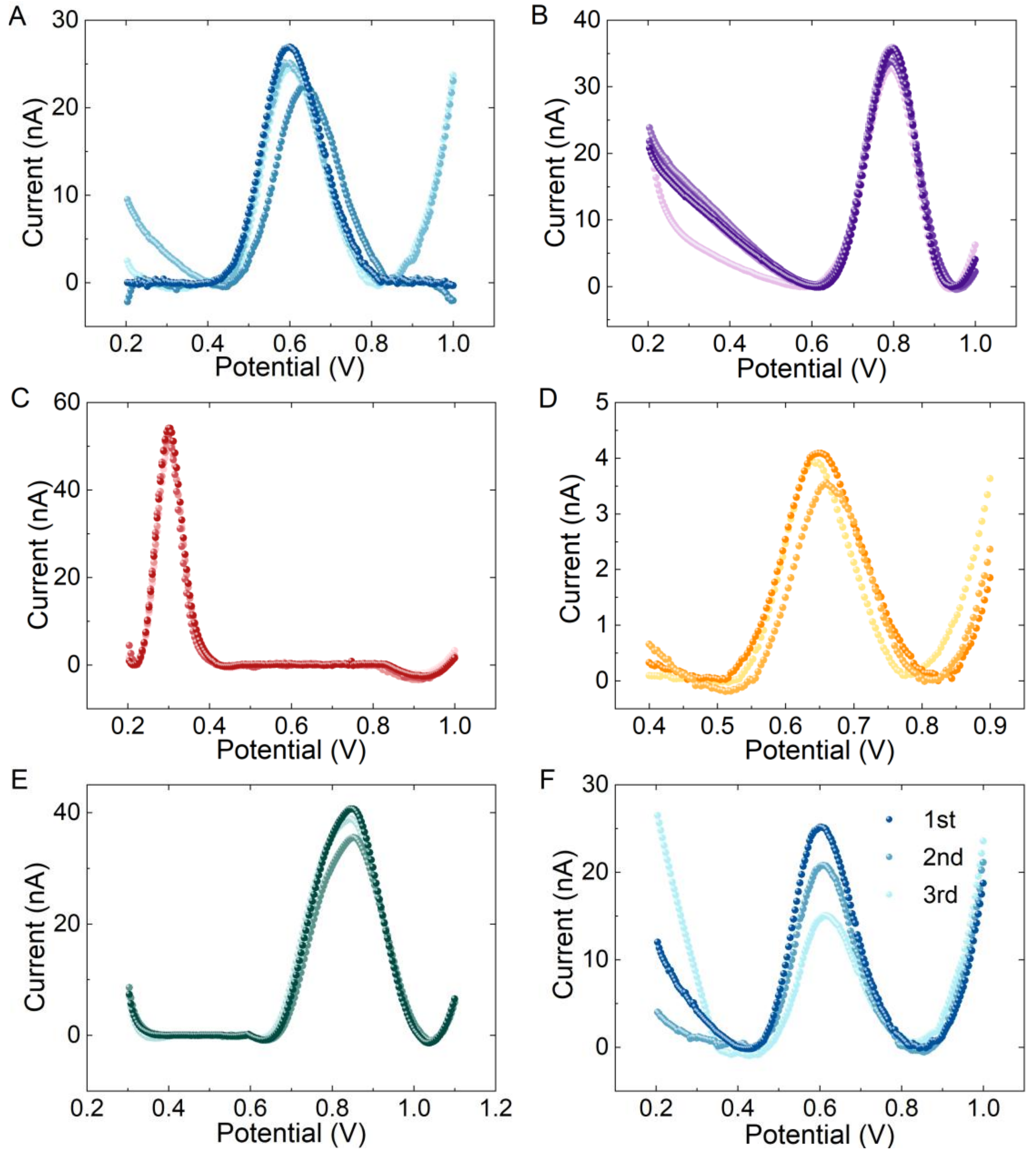

Supplementary Figure S5. Results of reproducibility and stability tests for the CS/CNF-Au electrode. (A–E) Reproducibility test results: (A) Tyrosine; (B) Tryptophan; (C) Norepinephrine; (D) Melatonin; (E) Tryptamine; (F) Stability test results for tyrosine.

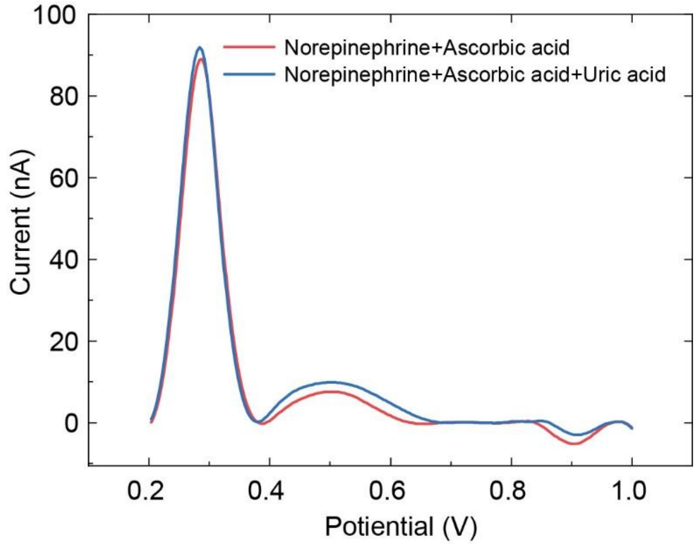

Supplementary Figure S6. The anti-interference performance of the CS/CNF-Au electrode. (1000 μM Norepinephrine; 1000 μM Ascorbic acid; 100 μM Uric acid)
